# Autophagy, apoptosis, vitamin D, and vitamin D receptor in hepatocellular carcinoma associated with hepatitis C virus

**DOI:** 10.1097/MD.0000000000010172

**Published:** 2018-03-23

**Authors:** Mohamed Ahmed Abdel-Mohsen, Ahlam Abd-Allah El-Braky, Abeer Abd El-Rahim Ghazal, Mohammed Mohammed Shamseya

**Affiliations:** a Department of Applied Medical Chemistry; b Department of Microbiology; c Department of Clinical and Experimental Internal Medicine, Medical Research Institute, Alexandria University, Alexandria, Egypt.

**Keywords:** apoptosis, autophagy, HCC, HCV, vitamin D, vitamin D receptor

## Abstract

The aims of this study were to investigate the interplay between autophagy and apoptosis and to investigate the association between both of autophagy and apoptosis and vitamin D and its receptor in hepatitis C virus (HCV) viral infection and its implication in the progression into hepatocellular carcinoma (HCC).

A cross-sectional study where serum levels of microtubule-associated protein 1A/1B-light chain 3 (LC3); marker of autophagy, caspase-3; marker of apoptosis, vitamin D3 and vitamin D receptor (VDR) were measured in healthy subjects as well as HCV and HCV-HCC patients using enzyme-linked immunosorbent assay technique.

Collectively, the liver profile revealed hepatic dysfunctions in HCV patients with or without HCC. A significant reduction in the serum concentration levels LC3 and caspase-3 were observed referring to the down regulation of autophagy and host-mediated apoptosis in HCV patients with or without HCC. Deficiency of vitamin D and decreased levels of its receptor were observed in HCV and HCV-HCC patients.

The perturbation in vitamin D/VDR axis, which modulates both of autophagy and apoptosis in HCV infection, may point out to its involvement and implication in the pathogenesis of HCV infection and the development of HCV-related HCC. Therefore, supplementation with vitamin D may not be the only solution to restore the vital biological functions of vitamin D but VDR-targeted therapy may be of great importance in this respect.

## 1 Introduction

Worldwide, hepatocellular carcinoma (HCC) is the sixth most common malignancy and the third most common cause of cancer death.^[1]^ In Egypt, liver cancer forms 1.68% of the total malignancies and HCC constitutes 70.48% of all liver tumors among Egyptians.^[2]^ One of the major causes of HCC is hepatitis C virus (HCV), which is considered to be the second most common cause of HCC, accounting for 25% of HCC cases.^[3]^ In Egypt, it has been shown that cirrhotic liver, owing to HCV infection, was observed in most of HCC patients.^[2]^

On the contrary, autophagy is a catabolic process with crucial roles in development, differentiation, homeostasis, and the survival of cells in nutrient-deprived conditions.^[4,5]^ Abundant evidence has revealed that autophagy is involved in the pathogenesis of various diseases such as liver diseases including viral hepatitis, fibrosis, cirrhosis, and HCC.^[4,6]^

Autophagy has been linked to other cell death pathways, for example, apoptosis and necrosis. Accumulating evidence reveals that autophagy and apoptosis can cooperate, antagonize or assist each other, thus influencing differentially the fate of the cell. It has delineated several pathways that mediate the complex interplay between autophagy and apoptosis providing mechanistic insight into the network that regulates both processes.^[7]^

On the contrary, it has been reported that autophagy can be regulated by vitamin D3 signaling at different levels, including induction, nucleation, elongation to maturation, and degradation. Also, the association between vitamin D3 and autophagy in innate immunity,^[8–10]^ inflammatory bowel diseases,^[11]^ infection, and cancer^[12,13]^ has been reported. Moreover, dysfunction of vitamin D receptor (VDR) and vitamin D deficiency can increase the risk of many chronic diseases, including infectious diseases and cancer.^[14]^

Accordingly, the present study was undertaken to investigate, in one hand, the relationship between autophagy and apoptosis and, on other hand, the association between autophagy and vitamin D and its receptor in HCV viral infection and its implication in the progression into HCC. The objectives of the present study were approached by assessing serum levels of LC3; as a marker of autophagy, caspase-3; as a marker of apoptosis, vitamin D3 and VDR in healthy subjects as well as HCV patients with or without HCC.

## 2 Subjects and methods

### 2.1 Subjects

The present study conforms to the ethical guidelines of the 1975 Declaration of Helsinki and was carried out after the approval of the Ethical Board Committee, Medical Research Institute, Alexandria University. Patients selected from those who were referred to Clinical and Experimental Internal Medicine Department, Medical Research Institute, Alexandria University in the period from January 2016 to end of March 2016 as reflected in a priori approval by the institution's human research committee. A signed informed consent was obtained from all subjects and patients enrolled in the present study who were divided into 2 groups: Group I, healthy subjects (HS), comprised of (30) apparently healthy subjects with no previous history of malignant or hepatic disease (negative for anti-HCV Abs and HBsAg); group II (HCV patients), comprised of (30) HCV-infected patients (positive for anti-HCV Abs and HCV-RNA); and group III (HCV-HCC patients), comprised of (30) HCC patients on top of HCV (positive for anti-HCV Abs and HCV-RNA) diagnosed clinically by 4-phase multidetector computed tomography or dynamic contrast enhanced magnetic resonance imaging.

### 2.2 Sampling

Venous blood samples were collected from all subjects and patients enrolled in the present study. Sera were separated, divided into aliquots and stored at −80°C until used.

### 2.3 Virological investigation

Detection of anti-HCV Abs and HBsAg was carried out routinely in Microbiology Department, Medical Research Institute, Alexandria University, using enzyme-linked immunosorbent assay (ELISA) technique (Abbott Murex Diagnostic Division).

Qualitative detection of HCV-RNA by reverse transcriptase PCR (RT-PCR) was carried out using QIAamp Viral RNA Mini Kit (Catalogue No 52904) according to the manufacturer's instructions.

### 2.4 Biochemical investigations

Activities of serum aminotransferases, aspartate aminotransferase (AST), and alanine aminotransferase (ALT), as well as serum levels of bilirubin and albumin were measured by colorimetric methods using Biosystems kits (Biosystems S.A./Costa Brava, Barcelona, Spain).

ELISA kit (MAPILC3A/LC3, Catalogue No 201-12-5438) for the determination of human serum LC3 was purchased from (Sun Red Biotechnology Company, Shanghai, China). ELISA kit (Caspase-3/Cpp32, Catalogue No 201-12-5438) for the determination of human serum Caspase-3 was purchased from (Sun Red Biotechnology Company). Thus, serum LC3 and caspase-3 levels were determined in accordance with the manufactures’ instructions.

ELISA Kit (Catalogue No 49–51) for the determination of human serum 25-OH Vitamin D3/D2 was purchased from (Orgentec Diagnostika GmbH, Mainz, Germany). Serum 25-OH Vitamin D3/D2 level was determined in accordance with the manufacturer's instructions.

ELISA Kit (Catalogue No 201-12-1554) for the determination of human serum VDR was purchased from (Sun Red Biotechnology Company, Shanghai, China). Serum 25-OH Vitamin D3/D2 receptor level was determined in accordance with the manufacturer's instructions.

### 2.5 Statistical analysis

Statistical analysis was performed with IBM SPSS statistics 20.0 for windows. The distribution of quantitative variables was tested by using of a Mann-Whitney *U* test, which was used to compare the mean level of biochemical parameter. Pearson (for abnormally distributed variables) correlation test was used to explore the association between variables. Generally, *P* <0.05 was considered to indicate a statistically significant difference.

## 3 Results

### 3.1 Liver functions profile

In HCV group, the mean activity levels of serum AST and ALT were significantly elevated when compared to that in apparently healthy subjects (*P* = .0001 and *P* = .015). Also, the mean serum concentration level of bilirubin was significantly increased in HCV patients when compared to that in apparently healthy subjects (*P* = .0001). The data showed a significant decrease in the mean concentration level of serum albumin of HCV patients when compared to that in apparently healthy subjects, (*P* = .0001), (Table 1).

**Table 1:**
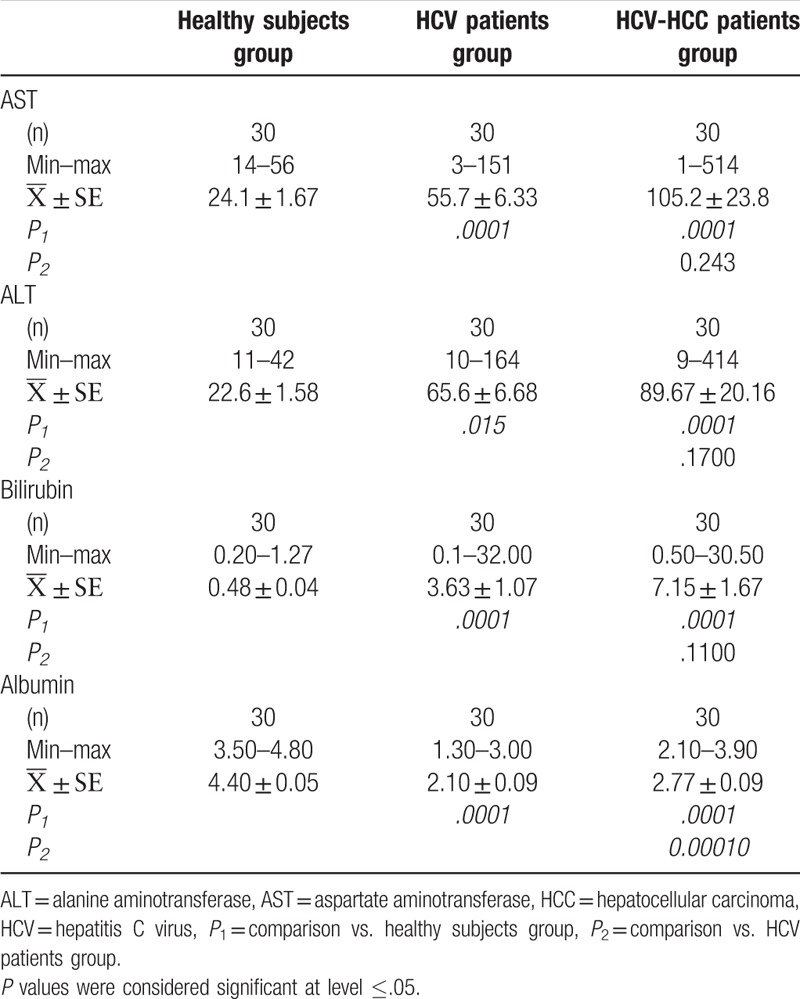
Statistical analysis of liver function tests of healthy subjects, HCV, and HCV-HCC groups.

In HCV-HCC group, the mean activity levels of serum AST and ALT and the mean concentration level of serum bilirubin were significantly higher when compared to that in apparently healthy subjects, *P* = .0001, *P* = .0001, and *P* = .0001, respectively. The mean concentration level of serum albumin in HCV-HCC patients was significantly lower than that in apparently healthy subjects (*P* = .0001, Table 1).

On the contrary, the mean concentration level of serum bilirubin and the mean activity levels of serum AST and ALT in HCV patients were not significantly different from those in HCV-HCC patients, *P* = .243, *P* = .170, and *P* = .110, respectively. Meanwhile, the mean concentration level of serum albumin in HCV patient was significantly lower than that in HCV-HCC patients, *P* = .0001, (Table 1).

### 3.2 Biomarkers of autophagy and apoptosis

**Figure 1:**
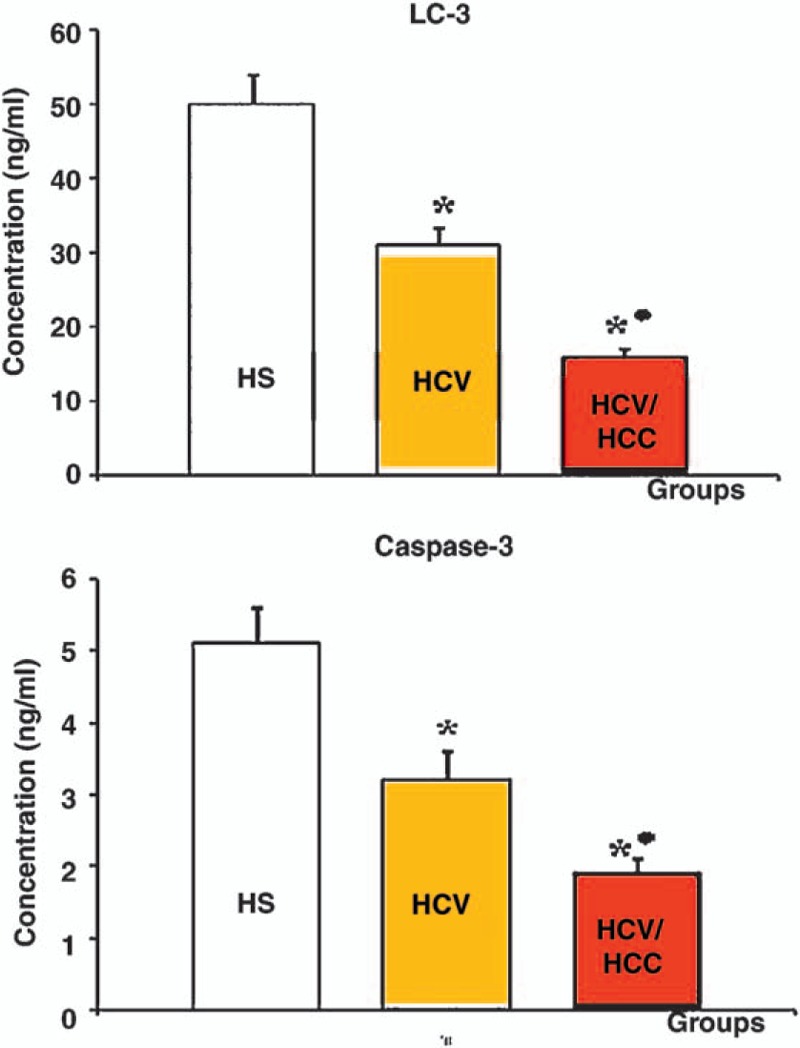
Mean serum concentration levels of biomarkers of autophagy, LC3, and apoptosis, caspase-3, in HS, HCV, and HCV-HCC Groups ^**∗**^ Statistically significant when compared to healthy subjects group **●**Statistically significant when compared to HCV patients group. *P* values were considered significant at level ≤.05. HCV = hepatitis C virus, HCV-HCC = hepatitis C virus-hepatocellular carcinoma, HS = healthy subjects.

In HCV and HCV-HCC patients, the concentration level of serum caspase-3 was significantly lower than that in apparently healthy subjects, *P* = .009 and *P* = .0001, respectively. This decrease in serum caspase-3 concentration level was more pronounced in HCV-HCC patients when compared to that in HCV patients, *P* = .003 (Fig. 1).

On the contrary, the mean serum concentration level of LC3 was significantly lower than that in apparently healthy subjects, *P* = .001 and *P* = .0001, respectively. Also, the mean concentration level of serum LC3 in HCC-HCV patients was significantly lower than that in HCV patients, *P* = .0001 (Fig. 1).

### 3.3 Vitamin D2/D3 and VDRs

**Figure 2:**
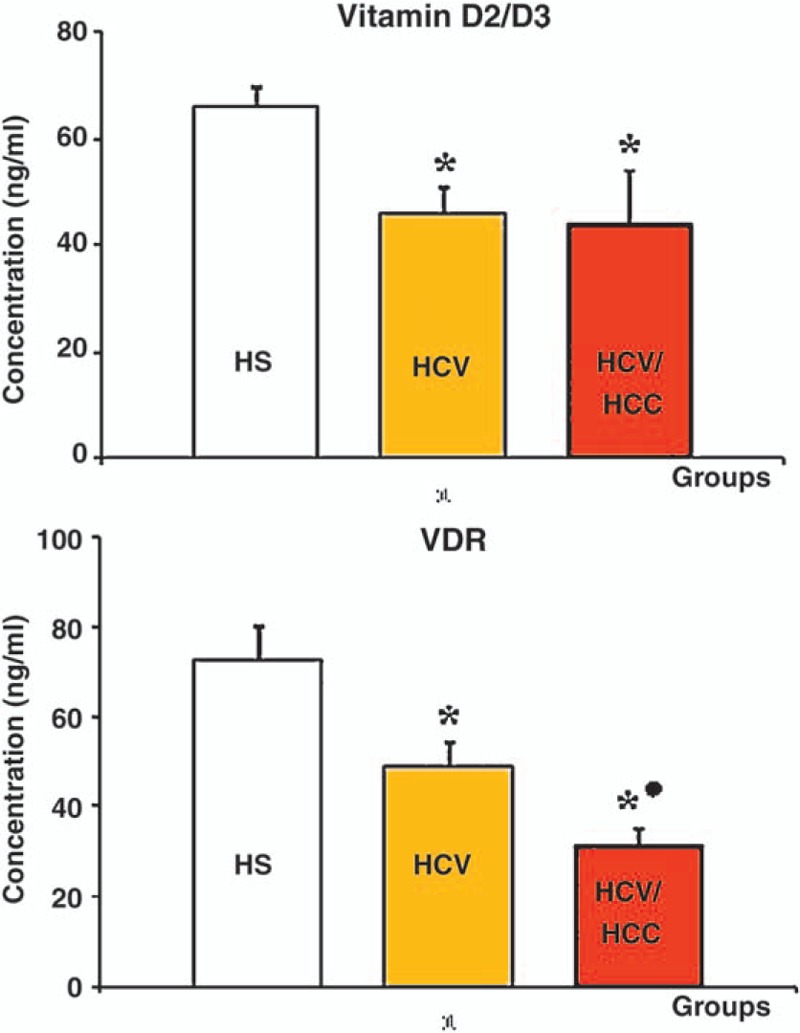
Mean serum concentration levels of vitamin D3/D2 and VDR in HS, HCV, and HCV-HCC groups.^**∗**^ Statistically significant when compared to healthy subjects group **●** Statistically significant when compared to HCV patients group. *P* values were considered significant at level ≤.05. HCV = hepatitis C virus, HCV-HCC = hepatitis C virus-hepatocellular carcinoma, HS = healthy subjects, VDR = vitamin D receptors.

In HCV and HCV-HCC patients, the mean concentration levels of Vitamin D2/D3 and its receptors were significantly lower when compared to that in apparently healthy subjects, *P* = .0001 and *P* = .0001, respectively (Fig. 2).

On the contrary, although the mean concentration level of serum Vitamin D2/D3 in HCC-HCV patients was insignificantly different from that HCV patients, *P* = .728, the mean concentration level of its receptor showed a significant decrease, *P* = 0.002 (Fig. 2).

### 3.4 Biostatistical correlations

**Figure 3:**
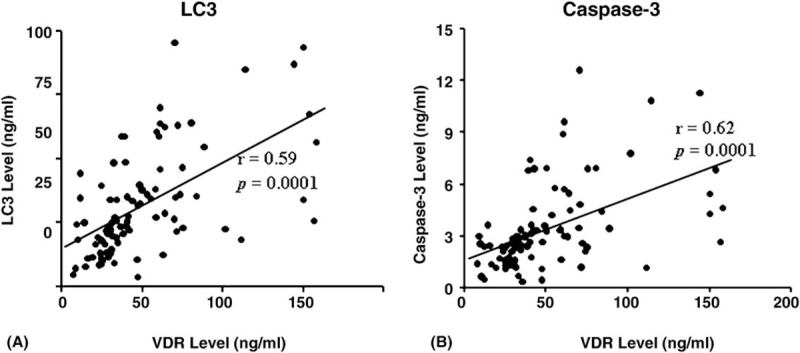
Overall correlation between serum level of VDR and (A) serum level of LC3 and (B) serum level of Caspase-3. *r* = Spearman correlation coefficient. *P* values were considered significant at level ≤.05.LC3 = light chain 3, VDR = vitamin D receptor

The result of the present study showed a statistically significant positive correlation between AST serum activity level and each of serum activity level of ALT (*r* = 0.57), serum concentration level of bilirubin (*r* = 0.67). Also, serum activity level of ALT was found to be significantly correlated to the serum concentration level of bilirubin in a positive manner (*r* = 0.42). On the contrary, a statistically significant negative correlation has been found between serum albumin concentration level and each of serum activity level of AST (*r* = −0.51), ALT (*r* = −0.38), as well as serum concentration level of bilirubin (*r* = −0.53) (Table 2). Moreover, the result of the present study showed a statistically significant positive correlation between VDR serum level and each of serum LC3 and caspase-3 levels (*r* = 0.59 and *r* = 0.62, respectively, Fig. 3).

**Table 2:**
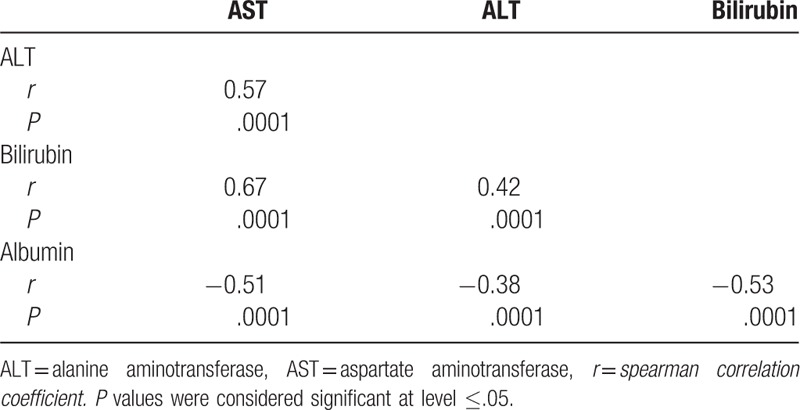
Overall correlation among liver function tests.

## 4 Discussion

The results of liver profile of patients enrolled in the present study (HCV and HCV-HCC patients) may point out to hepatic dysfunctions. In agreement with previous studies,^[15,16]^ in both of patients groups, HCV and HCV-HCC, a significant elevation in the activities of serum aminotransferases, AST and ALT, and serum bilirubin concentration level has been observed. The elevated levels of aminotransferases act as indicators of liver cell injury and are usually predominant in liver cirrhosis with increased ALT levels.^[17,18]^ Also, in HCC, elevated levels of aminotransferases may reflect damage to adjacent hepatocytes as a direct result of tumor growth or damage to more remote liver cells caused by interference with their blood supply or venous drainage. It may also be because of continuing liver cell necrosis in those with concomitant active cirrhosis or chronic active hepatitis.^[19]^ Moreover, high bilirubin level is usually associated with liver metastases and liver tumor involvement.^[20]^ On the contrary, a significant reduction in serum albumin level was noticed which could be because of chronic liver failure caused by cirrhosis.^[21]^

Serum levels of LC3, the most widely used monitoring biomarker for autophagy,^[22]^ were significantly reduced in HCV and HCV-HCC patients groups. To explore the role of autophagy in HCV infection, several studies were undertaken which were carried out on liver biopsies or infected liver cells in vitro.^[23]^ Therefore, the present study may be among the first studies to report the significant reduction in serum LC3 level of HCV-infected patients with or without liver cancer. Accordingly, these observations may point out the inhibition of autophagy in HCV patients with or without HCC.

El-Aggan et al, 2015^[24]^ have investigated the role of mammalian target of rapamycin (mTOR) and autophagy in the development and progression of HCV-related HCC. The results of their study showed that serum levels of mTOR, an autophagy inhibitor,^[25]^ were significantly elevated in cirrhotic HCV patients with or without HCC. Also, they showed that serum mTOR levels were significantly higher in patients with HCV-HCC than in HCV patients.^[24]^ These observations may be in consistence with the results of the present study and point out to the downregulation of autophagy in HCV patients with or without HCC. This observation together with observation that LC3 serum level was significantly lower in HCV-HCC patients than in HCV patients may explain the prounounced inhibition of autophagy in those patients. It has been proposed that the ability of HCV to inhibit the autophagic flux may depend on the viral genotype.^[26]^ It could be argued that HCV could control autophagy through modulating the activity of the cytosolic RNA-sensing protein kinase PKR, which has been reported to regulate virus-induced autophagy.^[27,28]^ The capability of HCV to either activates (via RNA IRES and core protein] or to inhibit PKR (via NS5A and E2 proteins) at different steps of the viral life cycle,^[27]^ could possibly account for a dual regulation of autophagy activity in the course of infection.

On the contrary, during HCV infection, apoptosis can be induced as a cellular defense mechanism mediated indirectly by immune attack of infected cells or directly by viral infection. However, HCV has evolved several ways to block host-mediated apoptosis. Accumulating evidence suggests that HCV proteins have the ability to inhibit host cell apoptosis.^[29–31]^ In this context, the results of the present study revealed the significant reduction in serum caspase-3 which, in turn, may reflect the inhibition of host-mediated apoptosis. Besides forming virus, viral core protein can regulate gene transcription, cell proliferation, apoptosis, and eventually progression to HCC.^[32,33]^ Zekri et al, 2011,^[34]^ have proposed several scenarios explaining how HCV infection can modulate apoptotic machinery pathway[s] during the course of infection. They claimed, as the disease progresses apoptosis is inhibited because of inactivation of caspases, upregulation of Bcl-2 family members, impairment in Bak gene expression, and increasing the expression of FasL. This signaling cascade favors cell survival with persistence of HCV infection and enhances the possibility of HCC development.

Serum 25-hydroxy vitamin D is the main circulating form of vitamin D and the most appropriate indicator of vitamin D status. It binds to its cognate receptor, VDR, which is a member of the nuclear receptor superfamily^[35]^ to exert its biological activities. The observed vitamin D deficiency in the present study is in agreement with previous studies, which have stated that vitamin D deficiency is very common in patients with chronic hepatitis C virus infection.^[36–38]^ Also, several studies have demonstrated the relationship between vitamin D status of patients with chronic hepatitis C and disease outcome.^[36,39–41]^ Moreover, and in addition to its deficiency in chronic HCV patients, an independent inverse relationship between vitamin D serum levels and the severity of liver fibrosis was described.^[42]^

It is worth mentioning that deficiency of vitamin D in chronic liver disease could be attributed in part to hepatic synthesis dysfunction which is evidenced by the fact that vitamin D deficiency is highly prevalent in noncirrhotic patients^[43]^ and the normalization of vitamin D level in cirrhotic patients after supplementation.^[44,45]^ Additionally, it has been shown that HCV reduces the production of 7-dehydrocholesterol, the precursor of endogenously produced vitamin D.^[46]^

On the contrary, several studies have reported downregulation of VDR expression in chronic hepatitis C.^[47–49]^ In the line of these studies, to our knowledge, the present study may be among the first studies to report the reduction in serum level of VDR in HCV patients with or without HCC. Thus, the reduction in both serum levels of vitamin D and its receptor may reflect the dysfunction of vitamin D/VDR signaling pathway. This dysfunction, in turn, will have its impact on the vital role of vitamin D as anti-inflammatory, anti-proliferative, and anti-tumourgenesis. Furthermore, the results of the present study may point out to the crucial role of VDR in vitamin D-mediated biochemical process including autophagy and apoptosis. Evidently, the results of the present study revealed a strong positive correlation between VDR and biomarkers of autophagy, LC3, and apoptosis, caspase-3. Such correlations were not observed between vitamin D and both biochemical markers referring to the indirect importance of VDR in such pivotal biochemical processes.

Wu and Sun, 2011,^[50]^ have reviewed the role of vitamin D signaling in autophagy homeostasis and demonstrated that its signaling can modulate autophagy, either increase or decrease, at several levels, throughout different mechanisms. The molecular mechanism that may explain the observed strong positive correlation between VDR and LC3 could as follow: vitamin D3 is a major regulator of calcium metabolism.^[51]^ Increased circulating vitamin D activates VDR, leading to increased intestinal calcium absorption.^[52]^ In target cells, calcium is released from the sarcoplasmic or endoplasmic reticulum to activate calcium-dependent kinases and phosphatases, thereby regulating numerous cellular processes, including autophagy. ER calcium induces autophagy when stimulated by vitamin D. This process is inhibited by mTOR, a negative regulator of autophagy, and induces massive accumulation of autophagosomes in a beclin-1- and ATG7-dependent manner as they are not fused with lysosomes.^[53]^ Vitamin D can downregulate the expression of mTOR protein, thus inducing autophagy by inhibiting the mTORC1 complex.^[54]^ Moreover, VDR regulates autophagy through p19INK4D, a cyclin-dependent kinase inhibitor. Vitamin D induces the expression of p19INK4D in SCC25 cells, thus protecting cells from autophagy-induced death. It is clear that vitamin D signaling increases p19INK4D, which in turn decreases autophagy and decreases VDR bound to the promoter of the p19INK4D gene.^[55]^ Thus, the dysfunction in vitamin D-VDR signaling pathway may, in turn, have its impact on autophagy and may lead to its inhibition. Apart from the involved molecular mechanism, it has been proposed that both vitamin D signaling and autophagy play a critical role in the pathogenesis of chronic inflammation and infection^[9]^ in addition to the development of HCV-related HCC.

In addition to the antiproliferative effects, there is increasing evidence that vitamin D exerts antitumor effects by regulating key mediators of apoptosis, such as repressing the expression of the antiapoptotic, prosurvival proteins BCL2, and BCL-XL, or inducing the expression of pro-apoptotic proteins, for example, BAX, BAK, and BAD.^[56]^ In addition, vitamin D might also directly activate caspase effector molecules, although it is unclear whether vitamin D-induced apoptosis is caspase-dependent^[57,58]^. Another possible mechanism in epithelial ovarian cancer cells for vitamin D-mediated apoptosis was proposed by Jiang et al, 2004,^[59]^ who showed that vitamin D destabilizes telomerase reverse transcriptase (TERT) mRNA, therefore inducing apoptosis through telomere attrition resulting from the downregulation of telomerase activity.

In conclusion, the present study may lead to the suggestion that molecular mechanisms of anti-autophagy and anti-apoptotic activity of HCV might play a pivotal role in regulation of hepatic cell growth and development of HCV-HCC and probably enabling the survival and growth of neoplastic hepatocytes. Additionally, the dysfunction of vitamin D/VDR axis which modulates both of autophagy and apoptosis in HCV infection may point out to its involvement and implication in the pathogenesis of HCV infection and the development of HCV related HCC. VDR may play a crucial role in vitamin D regulation of autophagy and apoptosis during the course of HCV. Therefore, impairment in the expression of VDR, indicated by its serum level, may comprise in vitamin D biochemical function as anti-inflammatory, antiproliferative, and antitumorgenesis. Therefore, supplementation with vitamin D may not be the only solution to restore the vital biological functions of vitamin D but VDR-targeted therapy may be of great importance with this respect.

### 4.1 Statement of human rights

All procedures performed in this study were in accordance with the ethical standards of the Ethical Committee (IORG0008812), Medical Research Institute, Alexandria University, and with the 1964 Helsinki declaration and its later amendments or comparable ethical standards.

### 4.2 Informed Consent

Informed consent was obtained from all individual participants included in this study.

## 5 Author contributions

**Methodology:** M. Shamseya.

**Supervision:** A.A-A. El-Braky, A.A.E.-R. Ghazal, M. Shamseya, M.A-M.A. Abdel-Mohsen.

**Writing – original draft:** A.A-A. El-Braky, A.A.E.-R. Ghazal, M. Shamseya, M.A-M.A. Abdel-Mohsen.

**Writing – review & editing:** A.A-A. El-Braky, A.A.E.-R. Ghazal, M.A-M.A. Abdel-Mohsen, M. Shamseya.
